# Expansion of the Genotypic and Phenotypic Spectrum of WASF1-Related Neurodevelopmental Disorder

**DOI:** 10.3390/brainsci11070931

**Published:** 2021-07-14

**Authors:** Siddharth Srivastava, Erica L. Macke, Lindsay C. Swanson, David Coulter, Eric W. Klee, Sureni V. Mullegama, Yili Xie, Brendan C. Lanpher, Emma C. Bedoukian, Cara M. Skraban, Laurent Villard, Mathieu Milh, Mary L. O. Leppert, Julie S. Cohen

**Affiliations:** 1 Department of Neurology, Boston Children’s Hospital, Harvard Medical School, Boston, MA 02115, USA; siddharth.srivastava@childrens.harvard.edu (S.S.); Lindsay.Swanson@childrens.harvard.edu (L.C.S.); David.Coulter@childrens.harvard.edu (D.C.); 2 Center for Individualized Medicine, Mayo Clinic, Rochester, MN 55902, USA; Macke.Erica@mayo.edu (E.L.M.); Klee.Eric@mayo.edu (E.W.K.); lanpher.brendan@mayo.edu (B.C.L.); 3 Department of Quantitative Health Sciences, Mayo Clinic, Rochester, MN 55902, USA; 4 GeneDx Inc., Gaithersburg, MD 20877, USA; smullegama@genedx.com (S.V.M.); yxie@genedx.com (Y.X.); 5 Department of Clinical Genomics, Mayo Clinic, Rochester, MN 55902, USA; 6 Roberts Individualized Medical Genetics Center, Children’s Hospital of Philadelphia, Philadelphia, PA 19104, USA; BedoukianE@chop.edu (E.C.B.); SkrabanC@chop.edu (C.M.S.); 7 Division of Human Genetics, Children’s Hospital of Philadelphia, Philadelphia, PA 19104, USA; 8 Department of Medical Genetics, AP-HM, La Timone Children’s Hospital, 13385 Marseille, France; laurent.villard@univ-amu.fr; 9 Inserm, Marseille Medical Genetics Center, Faculté de Médecine de Marseille, Aix Marseille University, 13385 Marseille, France; mathieu.MILH@univ-amu.fr; 10 Department of Pediatric Neurology, AP-HM, La Timone Children’s Hospital, 13385 Marseille, France; 11 Department of Neurology and Developmental Medicine, Kennedy Krieger Institute, Baltimore, MD 21205, USA; leppert@kennedykrieger.org; 12 Department of Pediatrics, Johns Hopkins University School of Medicine, Baltimore, MD 21287, USA; 13 Department of Neurology, Johns Hopkins University School of Medicine, Baltimore, MD 21287, USA

**Keywords:** *WASF1*, autism, intellectual disability, neurodevelopmental disorder

## Abstract

In humans, de novo truncating variants in *WASF1* (Wiskott–Aldrich syndrome protein family member 1) have been linked to presentations of moderate-to-profound intellectual disability (ID), autistic features, and epilepsy. Apart from one case series, there is limited information on the phenotypic spectrum and genetic landscape of WASF1-related neurodevelopmental disorder (NDD). In this report, we describe detailed clinical characteristics of six individuals with WASF1-related NDD. We demonstrate a broader spectrum of neurodevelopmental impairment including more mildly affected individuals. Further, we report new variant types, including a copy number variant (CNV), resulting in the partial deletion of *WASF1* in monozygotic twins, and three missense variants, two of which alter the same residue, p.W161. This report adds further evidence that de novo variants in *WASF1* cause an autosomal dominant NDD.

## 1. Introduction

*WASF1* (Wiskott–Aldrich syndrome protein family member 1)/WAVE1 (WASP-family verprolin homologous protein 1) forms one of the subunits of a protein complex (WAVE Regulatory Complex, WRC) that helps regulate actin remodeling. Within this pathway, binding of the GTPase RAC1 (Ras-related C3 botulinum toxin substrate 1) to distinct sites within the WRC alters the conformation of the complex, promoting accessibility of the VCA/WCA region within the complex to actin and the ARP2/3 (actin-related protein 2/actin-related protein 3) complex, which leads to actin filament assembly [1,2]. This process is necessary for neuronal roles like synaptic plasticity [3,4,5].

In humans, defects in *WASF1* have been associated with a neurodevelopmental disorder (NDD). In 2018, Ito et al. reported a case series of five unrelated individuals with moderate-to-profound intellectual disability (ID), autistic features, and epilepsy who had de novo truncating variants in *WASF1*. Three individuals shared a recurrent nonsense variant [c.1516C>T (p.R506*)], while the other two individuals had unique variants [c.1558C>T (p.Q520*) and c.1482delinsGCCAGG (p.I494Mfs*23)]. All these variants clustered around the WH2 (WASP homology 2) domain and were predicted to disrupt the C-terminal actin-binding WCA domain. In fibroblast samples from two individuals with the recurrent variant, functional studies revealed decreased production of the WASF1 protein as well as evidence of disrupted actin remodeling [6].

Apart from that case series, there is limited information on the phenotypic spectrum and genetic landscape of WASF1-related NDD. In this report, we describe in detail the developmental, neurological, and systemic findings of six individuals with WASF1-related NDD. We demonstrate a broader spectrum of neurodevelopmental impairment including more mildly affected individuals. Further, we report new variant types, including a copy number variant (CNV), resulting in partial deletion of *WASF1* in monozygotic twins, and three missense variants, two of which alter the same residue, p.W161. This report adds further evidence that de novo variants in *WASF1* cause an autosomal dominant NDD. 

## 2. Methods

All six patients (P1–P6) underwent clinical evaluations by a pediatric neurologist, medical geneticist, and/or developmental pediatrician. The patients had clinical genetic testing—including chromosomal microarray, exome sequencing, and, in some patients, single gene testing—as indicated based on phenotype.

The monozygotic twin sisters, P1 and P2, received a molecular diagnosis by chromosomal microarray, specifically a targeted oligonucleotide array comparative genomic hybridization (CGH), performed by the DNA Diagnostic Laboratory of Children’s Hospital Boston (Boston, MA, USA). DNA was extracted from peripheral blood, fragmented, labeled, and hybridized to the array CGH. The father and mother underwent targeted testing for the CNV identified in the sisters. P1 and P2 later underwent clinical exome sequencing, performed at GeneDx Laboratory (Gaithersburg, MD, USA), which did not identify the deletion nor any other contributory variants; however, CNV analysis was not performed as part of the exome sequencing analysis.

The other four patients, P3–P6, received a molecular diagnosis by trio exome sequencing performed in clinical diagnostic laboratories. Sanger sequencing by the diagnostic laboratories confirmed the detected *WASF1* variants. For P3–P5, exome sequencing was performed by GeneDx Laboratory (Gaithersburg, MD, USA) with methods as previously reported [7]. For P6, WES was performed using a TruSeq Exome kit (Illumina, San Diego, CA, USA) and sequenced on the Illumina NextSeq 500 (Illumina, San Diego, CA, USA) in Marseille Medical Genetics Center (MMG, Marseille, France). Data were analyzed using Varaft [8].

The patient cohort was assembled via connections through GeneMatcher [9].

## 3. Results

### 3.1. Demographics and Genotype

**Figure 1:**
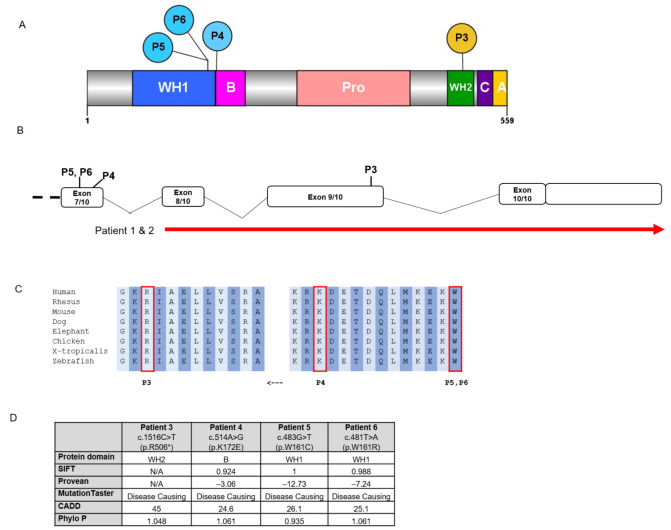
Schematics depicting location of *WASF1* variants and evidence of pathogenicity of the *WASF1* single nucleotide variants (SNVs). (**A**) Scheme 1 protein and the locations of the SNVs in P3–P6. Blue circles denote missense variants, and the yellow circle denotes the nonsense variant. Abbreviations of domains: WASP homology 1 domain (WH1, in blue), Basic domain (B, in magenta), Proline-rich region (Pro, in salmon pink), WASP homology 2 domain (WH2, in green), Cofilin homology domain (C, in purple), Acidic domain (A, in yellow). (**B**) Schematic diagram showing the 3′ end of *WASF1*. Red line indicates the deletion in P1 and P2, which includes exons 8–10 of *WASF1* and extends beyond the 3′ end of the gene. (**C**) Schematic diagram depicting conservation throughout various species of WASF1 amino acids including and surrounding the missense variants identified in this report. (**D**) Table summarizing protein domains and results of in silico pathogenicity prediction tools for the four single nucleotide variants, which are described in reference to RefSeq transcript NM_003931.2.

Among the six individuals in this cohort, four were unrelated and two were monozygotic twin sisters. All had de novo variants, consisting of a chromosomal deletion encompassing a portion of the *WASF1* gene in the twin sisters and *WASF1* single nucleotide variants (SNVs) in *n* = 4 (Table 1, Figure 1).

The chromosomal copy number variant (CNV) in the twin sisters was a deletion within cytogenetic band 6q21, approximately 180 kb in size, with genomic location as follows: arr[NCBI37/hg19] 6q21(110243269_110423466) × 1. The deleted interval involves two genes: the entire *GPR6* gene and exons 8–10 of *WASF1*. 

Among the four patients with SNVs, one individual (P3) had the recurrent nonsense variant c.1516C>T (p.R506*) previously published [6]. The remaining three individuals with SNVs had missense variants: c.514A>G (p.K172E) in P4; c.483G>T (p.W161C) in P5; and c.481T>A (p.W161R) in P6. Notably, two of the missense variants altered the same amino acid residue (p.W161), and all three clustered at or near the end of the WH1 (WASP homology 1) domain. Evidence of pathogenicity of the missense variants is shown in Figure 1. All sequence variants are described in reference to RefSeq transcript NM_003931.2.

### 3.2. Neurodevelopmental Features

All individuals in this cohort had global developmental delay (GDD) or ID (Table 2). One of the youngest individuals in the cohort (P4) had a diagnosis of GDD, though her visual-motor and language abilities were in the low-to-average range. She underwent a developmental assessment at age 32 months using the Clinical Adaptive Test/Clinical Linguistic and Auditory Milestone Scale (CAT/CLAMS) [10], which demonstrated a visual motor/problem-solving developmental quotient of 77% and a language developmental quotient of 72%. At this age, she was able to jargon and use 50–100 words as well as two-word phrases. Among the remaining five participants, best estimate of ID severity (based on factors such as adaptive skills) ranged from moderate ID in *n* = 1 to severe-to-profound ID in *n* = 4. None of these five individuals had spontaneous, specific spoken words. Notably, there was no history of developmental regression in any of the participants. 

Behavioral challenges in the cohort included a nearly universal presence of autism spectrum disorder (ASD). Half (*n* = 3/6) exhibited repetitive hand movements, including midline hand stereotypies and hand-wringing behaviors. These behaviors prompted consideration of Rett syndrome, specifically *MECP2* gene sequencing in P1, P2, and P5, and *CDKL5* gene sequencing in P5; prior to exome sequencing, the results were normal. Anxiety was noted in *n* = 1/6, and aggressive behaviors occurred in *n* = 3/6. 

Motor challenges were common in this cohort. All individuals had hypotonia, and one individual had spasticity and dystonia. From a functional motor standpoint, the most severely affected individual was P6, who was only just starting to crawl at age 4 years. The twin sisters also had severe motor limitations, as neither could walk independently for long distances. By comparison, the other three individuals were able to walk independently, but age of achievement of this milestone was delayed, ranging from 2 to 3 years. 

### 3.3. Neurological Features

Among neurological features, epilepsy affected *n* = 5/6 individuals. Seizure types included generalized tonic clonic seizures (*n* = 1), tonic seizures (*n* = 2), focal seizures (*n* = 1), reflex seizures (*n* = 1), and myoclonic seizures (*n* = 1). Infantile spasms occurred in P6. Among the individuals with epilepsy in the cohort, electroencephalogram (EEG) showed evidence of slowing, as well as focal and generalized epileptiform activity. Brain MRIs were normal in *n* = 3 patients and showed non-specific abnormalities in *n* = 3 patients. 

### 3.4. Systemic Features

None of the patients had congenital anomalies or consistent dysmorphic features (Table 3). Some had variable additional systemic manifestations. Notable growth issues included failure to thrive (*n* = 2/6) and short stature (*n* = 2/6). Endocrine features consisted of growth hormone deficiency (*n* = 1/6), hypothyroidism (*n* = 1/6), and precocious puberty (*n* = 1/6). Strabismus was present in 3/6 of the individuals, including both twin sisters, one of whom also had optic atrophy. Vasomotor instability occurred in the twin sisters. Except for the twin sisters who were born at 26 weeks gestation, the perinatal histories of the affected individuals were overall uncomplicated. 

## 4. Discussion

In this work, we expanded the genetic landscape of WASF1-related NDD to include de novo missense variants and a partial gene deletion. We report one patient with the recurrent p.R506* variant, bringing the number of published patients with this specific variant to four. We also broadened the phenotypic spectrum to include more mildly affected individuals. 

Multiple lines of evidence support our assertion that the three missense variants are causative of the patients’ phenotype, even though functional studies were not performed to confirm their pathogenicity and determine their mechanism of action (i.e., loss of function vs. gain of function vs. dominant-negative action). These variants are absent from general population databases, alter highly conserved amino acid residues, and are predicted by in silico tools to be deleterious. These variants are clustered in a known functional domain; two of the variants alter the same amino acid residue (p.W161), while the remaining variant (p.K172E) affects a residue that is nearby. Furthermore, the individuals with these variants in our cohort have a shared phenotype consistent with WASF1-related NDD (GDD/ID and seizures without congenital anomalies or major dysmorphic features).

We report a CNV causing WASF1-related NDD. The CNV is a deletion that affects exons 8–10 of *WASF1*, likely disrupting the C-terminal actin-binding WCA domain, which is also affected by the recurrent p.R506* variant. Of note, the CNV deletion affects not only exons 8–10 of *WASF1*, but also the entirety of *GPR6*, which encodes G protein-coupled receptor 6. Variants in *GPR6* have not been reported in association with human disorder to date. Based on gnomAD (https://gnomad.broadinstitute.org/, accessed on 27 May 2021) constraint metrics, *GPR6* is relatively tolerant to loss-of-function variation as evidenced by the probability of a loss-of-function intolerance (pLI) score of 0.07, which suggests that *GPR6* is likely tolerant to loss-of-function variation such as a heterozygous deletion [11]. In contrast, the pLI for *WASF1* is 1, indicating that it is highly intolerant to loss-of-function variation. The twin sisters’ phenotype is consistent with WASF1-related NDD, so most likely the partial deletion of *WASF1* accounts for their full phenotype, even though we cannot exclude the possibility that haploinsufficiency of *GPR6* is contributory. 

We broadened the severity spectrum of WASF1-related NDD to include individuals with milder cognitive impairment without epilepsy. All five individuals in the Ito cohort were severely affected: the highest level of intellectual functioning among them was moderate to severe ID (seen in *n* = 2), and the majority (*n* = 3) had severe to profound ID; moreover, all but one had epilepsy [6]. In contrast, one individual in our cohort (P4) was mildly affected in terms of cognitive impairment. On developmental testing at 32 months of age, her language and visual motor/cognitive developmental quotients were both above 70%. Assuming a continuation of this current developmental trajectory, these scores may suggest a trajectory towards mild ID or low-to-average IQ, rather than moderate-to-profound ID seen with the others in our cohort and in previously published cohort. Intriguingly, this patient has no current evidence of seizures, including normal EEG, though larger numbers of individuals are needed to determine if the presence of epilepsy within WASF1-related NDD confers a higher likelihood of more severely affected cognitive outcomes. 

Our report confirms that WASF1-related NDD is primarily associated with neurodevelopmental impairment but not major congenital anomalies or consistent dysmorphic features. Three of individuals in our cohort were non-dysmorphic, while the others had variable minor dysmorphisms. Relatively more common systemic manifestations included strabismus and growth/endocrine abnormalities (short stature, failure to thrive, precocious puberty), but none of the individuals presented with major structural anomalies. 

Rett syndrome-like features, particularly midline hand stereotypies/hand-wringing behavior, were present in half the cohort. Ito et al. referred to specific gene testing conducted among their cohort prior to the WASF1-related diagnosis, and *MECP2*, *CDKL5*, and *FOXG1* were among these considerations [6]. Altogether, these data suggest that WASF1-related NDD may be considered in the differential of individuals with Rett syndrome-like presentation, broadening the number of genes that can present as Rett mimics [12] and raising the idea that *WASF1* could be considered for inclusion in Rett syndrome gene sequencing panels. 

Motor impairment, including hypotonia, was universal in the cohort. In fact, half the cohort was unable to walk independently, while the other half had significant delays in achievement of this milestone (ranging from 2–3 years of age). These findings are similar to those seen in in the Ito cohort, in which age at walking ranged from 25 months to 10 years, and one individual was non-ambulatory at age 23 years [6]. 

In fact, the diagnosis of cerebral palsy (CP) could be applied to several of the reported patients with WASF1-related NDD, particularly the hypotonic CP subtype. This label, which has elicited controversy as a subtype of CP, refers to CP in which the predominant motor feature is hypotonia [13]. Broadly, CP is defined as a group of disorders of the development of movement and posture due to non-progressive injury to the developing brain [14]. The diagnosis of CP is agnostic to the etiology, which could include genetic and/or non-genetic factors. Notably, the twin sisters had several risk factors for CP including prematurity at 26 weeks and intraventricular hemorrhage, which could lead one to conclude that their impairments were due to perinatal brain injury. However, several characteristics prompted consideration of a genetic etiology: dysmorphisms, Rett-like features, and normal brain MRI in one of the sisters. The presence of a genetic disorder as the etiologic diagnosis should not preclude or negate a clinical diagnosis of CP, because a diagnosis of CP is based on clinical features not underlying etiology [15]. Genetic etiologies should be considered for patients with CP when certain indicators are present, such as lack of history of perinatal brain injury, presence of dysmorphic features or congenital anomalies, or mismatch in clinical presentation with what would be expected based on brain MRI and/or perinatal history [16]. 

## 5. Conclusions

In sum, WASF1-related NDD can present as a broad phenotypic spectrum ranging from mild-to-profound GDD/ID with variable features of ASD, epilepsy, and motor impairment. WASF1-related NDD may be caused by different variant types including missense variants and partial gene deletion. Further study is needed to delineate functional impact of missense variants. The small number of cases limits our ability to make genotype–phenotype correlations.

## Figures and Tables

**Table 1:** Demographic and genetic features of the cohort.

	P1 (MZ Twin)	P2 (MZ Twin)	P3	P4	P5	P6
Sex	Female	Female	Male	Female	Female	Male
Age at last exam	15 years	15 years	7 years	4 years 7 months	6 years	4 years
WASF1 variant previously reported	No	No	Yes	No	No	No
Basis for WASF1 variant discovery	Chromosomal microarray	Chromosomal microarray	Trio exome sequencing	Trio exome sequencing	Trio exome sequencing	Trio exome sequencing
WASF1 variant *	Partial gene deletion (exons 8–10)	Partial gene deletion (exons 8–10)	c.1516C>T	c.514A>G	c.483G>T	c.481T>A
Protein change	N/A	N/A	p.R506 *	p.K172E	p.W161C	p.W161R
Inheritance	De novo	De novo	De novo	De novo	De novo	De novo
Zygosity	Heterozygous	Heterozygous	Heterozygous	Heterozygous	Heterozygous	Heterozygous
Chromosomal microarray	As above	As above	609 kb duplication of 4q22.3, paternally inherited, likely benign	Normal	419 kb duplication of 4q12, paternally inherited, likely benign	Normal
Consanguinity	No	No	No	No	No	No

* All sequence variants are described in reference to RefSeq transcript NM_003931.2. N/A = not applicable; MZ = monozygotic.

**Table 2:** Neurological and developmental features of the cohort.

Language/Cognitive	P1 (MZ Twin)	P2 (MZ Twin)	P3	P4	P5	P6
Current best language abilities	Babbling	Babbling	Non-specific vocalizations	50–100 words; two-word phrases	Non-specific vocalizations	Non-verbal
Age of saying first word besides mama/dada	N/A	N/A	N/A	17 months	N/A	N/A
Age of speaking full sentences	N/A	N/A	N/A	3–4 years	N/A	N/A
Global delay (GDD)/intellectual disability (ID)	Yes (ID)	Yes (ID)	Yes (ID)	Yes (GDD)	Yes (ID)	Yes (ID)
IQ estimate	Profound ID	Profound ID	Moderate ID	Low-average (CAT/C:AMS at 32 months: language DQ 72%, visual motor/cognitive DQ 77%)	Severe ID	Profound ID
**Behavioral/Mood**						
Autism	Yes	Yes	Yes	Yes	Yes	No
Specific repetitive behaviors	Repetitive hand movements	Midline hand stereotypies	Head banging, hitting		Hand wringing	
Anxiety	No	Yes	No	No	No	No
Aggression	Yes	No	Yes	No	Yes (when frustrated)	No
**Motor**						
Current best motor abilities	Taking steps with support	Walking without support for a short number of steps	Walking independently	Walking independently	Climbing up steps with alternating feet	Starting to crawl
Age of walking independently	N/A	N/A	2 years	2 years 1 month	3 years	N/A
Axial hypotonia	Yes	Yes	Yes	Yes	Yes	Yes
Appendicular hypertonia	Yes (spasticity/dystonia)	No	No	No	No	No
**Neurological**						
Microcephaly	Yes	Yes	No	Borderline (6th percentile)	No	Yes
Cortical visual impairment	Yes	Yes	No	No	Yes (improving)	Possible (no eye tracking)
Epilepsy	Yes	Yes	Yes	No	Yes	Yes
Seizure types	Generalized tonic seizure	Tonic seizures with atonic components	Focal seizures, reflex seizures	N/A	Myoclonic seizures	Infantile spasms, tonic seizures, hypermotor seizures with dystonic postures
Disrupted sleep	Yes	Yes	No	No	Yes	No
Dysphagia	No	No	No	Yes	Yes (resolved)	Yes
Age of latest MRI brain	15 months	3 years	3 years	2 years 3 months	4 years	18 months
Brain MRI findings	Porencephalic cyst (sequela of prior intraventricular and intraparenchymal hemorrhage)	Normal	Mild, stable ventriculomegaly	Normal	Thickened anterior corpus callosum	Normal
Age of latest EEG	15 years	14 years	7 years	1 years 10 months	7 years	3 years
EEG findings	Diffuse slowing	Diffuse slowing Frontal spikes in sleep	Moderate slowing (bilateral midline and central regions, maximal left); focal epileptiform discharges (left midline, central head regions)	Normal	Diffuse slowing; generalized interictal epileptiform discharges (bifrontal predominance and associated frontal slowing); frequent myoclonic seizures (head jerks)	Slowing; slow paroxysmal abnormalities (bi-occipital location)

N/A = not applicable; MZ = monozygotic; DQ = developmental quotient; MRI = magnetic resonance imaging; EEG = electroencephalogram.

**Table 3:** Perinatal and systemic features of the cohort.

Perinatal Features	P1 (MZ Twin)	P2 (MZ Twin)	P3	P4	P5	P6
Method of conception	In vitro fertilization	In vitro fertilization	Unassisted	Unassisted	Unassisted	Unassisted
Pregnancy complications	Concern for twin-twin transfusion	Concern for twin-twin transfusion	None	None	Single umbilical artery, concern for small for gestational age	None
Gestational age	26 weeks	26 weeks	41 1/2 weeks	38 weeks	39 weeks	40 weeks
Delivery method	C-section	C-section	C-section	Vaginal	C-section	Vaginal
NICU stay?	Yes	Yes	No	No	No	No
Perinatal complications	Neonatal depression, intraventricular hemorrhage, intraparenchymal	Intraventricular hemorrhage	None	None	None	Difficulty breastfeeding
Birth weight	645 g	Unknown	4100 g	3260 g	3005 g	3690 g
Birth head circumference	Unknown	Unknown	36 cm	34 cm	Unknown	36 cm
Birth length	Unknown	Unknown	53.3 cm	50 cm	44.5 cm	49 cm
**Systemic Features**						
Dysmorphisms	Triangular face, midface hypoplasia, upslanting palpebral fissures, pointed chin	Triangular face, midface hypoplasia, upslanting palpebral fissures	Long face, simple ears	None	Frontal bossing, broad forehead, normal nasal bridge with squared tip	None
Growth	Normal	Normal	Failure to thrive	Short stature (parents had short stature)	Short stature	Failure to thrive
Endocrine	None	None	None	None	Hypothyroidism, growth hormone deficiency	None
Ophthalmological	Strabismus, optic atrophy	Strabismus	Strabismus, exotropia	None	Exotropia	None
Gastrointestinal	None	None	Constipation, failure to thrive	None	None	Gastrostomy at 3 years old
Musculoskeletal	Camptodactyly		Pes planus		In-toeing, tight heel cords	
Dermatological	Café au lait macules, lentiginous compound nevus	None	None	None	Stork bite	None
Autonomic	Vasomotor instability	Vasomotor instability	None	None	None	None

MZ = monozygotic.

## Data Availability

The data presented in this study is contained within the article.
